# Coracoclavicular fixation techniques for Neer IIb and “extralateral” fractures of the distal clavicle: a systematic review

**DOI:** 10.1016/j.xrrt.2021.06.007

**Published:** 2021-08-12

**Authors:** Andreas Panagopoulos, Konstantina Solou, Marios Nicolaides, Ioannis K. Triantafyllopoulos, Antonis Kouzelis, Zinon T. Kokkalis

**Affiliations:** a Department of Shoulder & Elbow Surgery, Patras University Hospital, Patras, Greece; b Division of Orthopaedics, Barts and The London School of Medicine and Dentistry, Queen Mary University of London, London, UK; c Hygeia Private Hospital, Athens, Greece

**Keywords:** Unstable extralateral distal clavicle fractures, Neer type IIb, Neer type IIc, Coracoclavicular fixation

## Abstract

**Background:**

Unstable “extralateral” fractures of the distal clavicle (lateral to the coracoclavicular ligaments) are not distinguished in the Neer classification system and are commonly included with Neer IIb fractures. In the literature, there is no optimal surgical technique for managing unstable fractures of the distal clavicle, nonetheless for unique “extra-lateral” patterns. The aim of this study is to evaluate the effectiveness and safety of existing coracoclavicular fixation techniques for managing unstable Neer IIb and extralateral (IIc) fractures of the distal clavicle.

**Methods:**

We performed a systematic search of the literature to capture all studies evaluating the safety and effectiveness of existing coracoclavicular loop techniques for unstable Neer IIb and extralateral (IIc) fractures of the distal clavicle. We searched the PubMed (MEDLINE and PubMed Central), Scopus, Web of Science, Google Scholar, and Cochrane Central Register of Controlled Trials electronic databases to retrieve studies published between January 2000 and November 2020. Our study was guided by a prospectively developed protocol and reported in accordance with the latest Preferred Reporting Items for Systematic Reviews and Meta-Analyses guidelines.

**Results:**

Our database search yielded a total of 564 records; of which, 21 were deemed appropriate for inclusion in our qualitative synthesis. The total number of reported IIb/c fractures managed with a coracoclavicular stabilization technique in all studies was 421. In total, 139 (33%) patients received arthroscopic-assisted treatment, and 282 (67%) patients were managed with open coracoclavicular stabilization. The reported clinical results were very good to excellent in most studies, whereas the overall major and minor complication rate was 2.6% and 12.8%, respectively. Major complications were more frequent in arthroscopic-assisted techniques (4.3%) compared with open (1.8%).

**Conclusion:**

The present systematic review of coracoclavicular stabilization techniques for unstable Neer IIb and extralateral fractures of the distal clavicle demonstrates promising clinical outcomes, including effectiveness and safety. We support the previously proposed modification of the Neer classification to include this unique type of unstable extralateral fracture (type IIc) to allow for targeted surgical management.

Clavicle fractures account for 2.6%-4% of all adult fractures, with the distal third being involved in 10%-30 % of cases.^1^ Given that up to 50% of distal clavicle fractures are displaced and 10%-44% can lead to symptomatic nonunion or malunion, precise classification is paramount to guide surgical management.^2^ Distal clavicle fractures are usually classified into 3 types as per Neer’s classification^3^ – a system that has been widely used since the 1960s. Type I is an undisplaced fracture lateral to coracoclavicular ligaments, type IIa is located medial to coracoclavicular ligaments, type IIb between the coracoclavicular ligaments, and type III is an intra-articular fracture. Graig^4^ later added types VI (epiphyseal fractures in children) and V (avulsed inferior cortical fragment attached to the coracoclavicular ligaments). However, these classifications do not include small fractures lateral to torn coracoclavicular ligaments with displacement of medial clavicle (“extralateral” type). Cho et al^5^ were the first to suggest classifying these as type IIc distal clavicle fractures, whereas they also proposed that type IId can replace type V. Nonetheless, Neer types IIa/IIb and V (or Cho types IIb to IId) are usually unstable and indicative of internal fixation.^6^

In the literature, there is no optimal surgical technique for managing unstable fractures of the distal clavicle, nonetheless for unique patterns such as IIc. Several surgical techniques have been described and are generally distinguished in two main categories; either rigid internal fixation (T-, locking-, hook-, double-plates, coracoclavicular or acromioclavicular screws, and intramedullary fixation) or flexible osteosynthesis with or without arthroscopic assistance (tension band wiring, sutures and bone anchors, tapes, cortical buttons, and synthetic grafts or allografts), as well as various combinations of these techniques.^7^, ^8^, ^9^, ^10^, ^11^ The evidence is even more vague for extr-lateral (Cho IIc) fractures, where the lateral clavicle fragment being relatively small, and is not always amenable to hold traditional hardware. Levy et al were the first to report on fractures classified as type IIc – of 48 patients enrolled in the study, 30 were treated with simple coracoclavicular suture stabilization and the rest underwent plate fixation and coracoclavicular augmentation – and conclude that there is a need for modifying the original Neer’s classification to include type IIc fractures.^12^

The aim of this study is to perform a systematic search of the literature to identify all studies evaluating the effectiveness and safety of coracoclavicular fixation techniques for managing Neer IIb and extralateral (Cho IIc) distal clavicle fractures in skeletally mature patients.

## Methods

We performed a systematic review of the literature in accordance with the Preferred Reporting Items for Systematic Reviews and Meta-Analyses Statement of 2009^13^ and the Cochrane Handbook for Systematic Reviews of Interventions.^14^ Our study was guided by a prospectively developed protocol outlining our methodology.

### Inclusion criteria

We aimed to capture randomized or quasirandomized controlled trials, cohort or case-control studies, and case series evaluating coracoclavicular surgical fixation techniques for the management of isolated distal clavicle fractures in skeletally mature patients. Studies were included only if fractures were either between the coracoclavicular ligaments with conoid being torn and trapezoid intact (Neer IIb) or lateral to the coracoclavicular ligaments with both being torn (extralateral, or Cho IIc). Studies were only reviewed if the published manuscript was in English, French, German, and Spanish. We included only studies that reported both efficacy and safety, measured by objective clinical scores and complication rates, respectively. We excluded any studies which did not meet the aforementioned criteria, studies on animals, and studies that reported on less than 5 fractures. Furthermore, we excluded studies which recruited solely participants younger than 16 years of age and studies that evaluated other treatment modalities (eg, hook plates, locking or other types of plates, transacromial or intramedullary fixation). We did not exclude comparative studies of two or more different surgical modalities, given that at least one was a coracoclavicular stabilization technique.

### Study identification and selection

We performed a comprehensive search of the literature on 15 November 2020. We searched the PubMed (MEDLINE and PubMed Central), Scopus, Web of Science, Google Scholar, and Cochrane Central Register of Controlled Trials electronic databases to retrieve studies published between January 2000 and November 2020. Our search strategy included a combination of keyword terms including “distal AND clavicle AND fracture” and “lateral AND clavicle AND fracture.” We supplemented the electronic database search by screening the bibliographies of relevant published studies and searching online registries for ongoing clinical trials (Clinical Trials Gov., ISRCTN, EU Clinical Trial Register).

We exported all captured studies into a reference manager library (EndNote X9) and removed all duplicates. The results were screened by two independent reviewers at two levels: title-abstract and full-text screening. We resolved any discrepancies during title-abstract screening stage by including the article by default and during full text screening by discussion and senior author consensus.

### Data collection

All relevant data were extracted using piloted forms and exported to a digital spreadsheet (Microsoft Excel). Data extraction was performed by two independent reviewers. We classified extraction fields into four main categories: study characteristics and methods, population demographics, surgical intervention, and outcomes and results. Any discrepancies in the extracted data were resolved by thoroughly inspecting the manuscripts during reviewer meetings.

### Risk of bias assessment

Our review did not capture any randomized controlled trials, thus the ROBINS-I tool for assessing risk of bias in nonrandomized studies was used.^15^ We stratified the risk for confounding, selection of participants into the study, classification of interventions, deviations from intended interventions, missing data, measurement of outcomes, selection of the reported result, and overall bias. Overall risk of bias was considered low risk if all domains were determined at low risk; moderate risk if at least one of the domains was determined at moderate risk but none as serious; serious risk if at least one of the domains was determined at serious risk but none as critical; and critical risk if at least one of the domains was determined at critical risk.

### Data synthesis and analysis

We synthesized all studies qualitatively using descriptive statistics, where applicable. We categorized the reported surgical techniques in 2 types: arthroscopic assisted coracoclavicular stabilization with buttons, with or without interfragmentary sutures or tension band, and open coracoclavicular stabilization with buttons, subcoracoid sutures, mersilene tapes, cables, or suture anchors. We reported findings for two main outcomes: effectiveness and safety. Effectiveness was evaluated by synthesizing and summarizing data from objective clinical scores as reported by the included studies. Safety was assessed by calculating the frequency of complications – we categorized complications into major (new fracture, implant failure, nonunion, coracoid fracture, deep infection) and minor (delayed union, hardware irritation, superficial infection, scar problems, mild osteolysis or button subsidence, malunion, slight loss of reduction, adhesive capsulitis).

## Results

**Figure 1:**
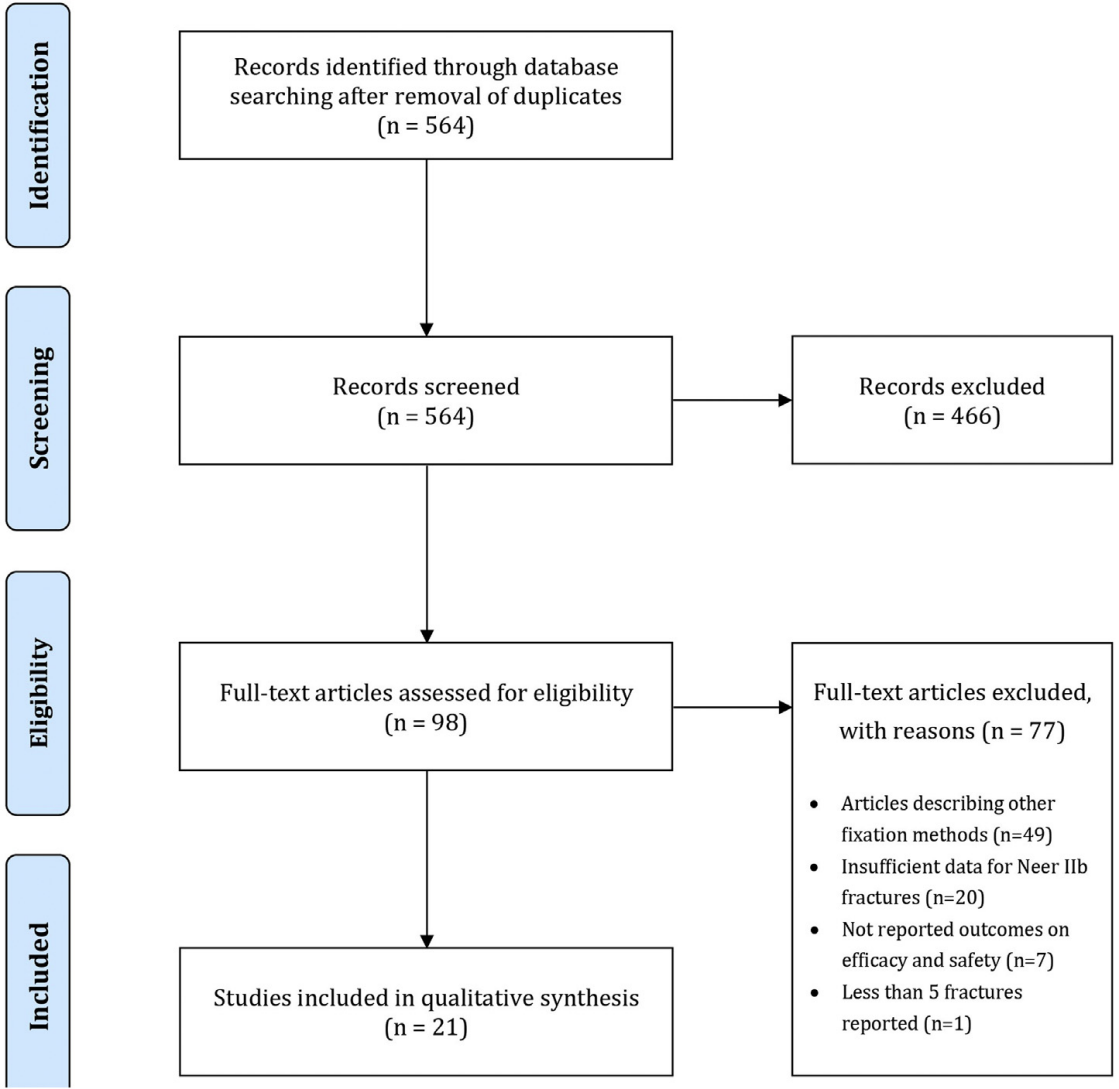
PRISMA flow diagram of study selection. *PRISMA*, Preferred Reporting Items for Systematic Reviews and Meta-Analyses.

Our database search after duplicate removal yielded a total of 564 records. We retrieved the full texts of 98 manuscripts to screen in their entity, whereas 466 records were excluded (Fig. 1). Only 21 were deemed appropriate for inclusion in our qualitative synthesis (Table I). The most common reason for exclusion was studies reporting data on management methods other than coracoclavicular surgical fixation techniques (n = 49) (Fig. 1).

**Table I:** Summary of key characteristics of included studies that report data on Neer IIb/Cho IIc fractures managed with a coracoclavicular stabilization surgical technique.

Author (country)	Yr	Study design	Neer IIB/Cho IIC	Mean age (yr)	Male/female	Surgical technique	Follow-up (mo)
Chen et al^5^ (Taiwan)	2002	Retrospective	11	37	8/3	CC stabilization with Mersilene tape and interfragmentary wire	27
Shin et al^29^ (Korea)	2009	Retrospective	19	43.4	14/5	Two suture anchors fixation augmented with fragment suture tension band	4.6
Li et al^19^ (China)	2011	Retrospective	29	34	21/8	Open CC stabilization with 2 titanium cables (drill hole in the coracoid)	32
Yang et al^35^ (Taiwan)	2011	Retrospective	28	37.9	18/10	Open CC stabilization (mersilene tape)	57.3
Takase et al^32^ (Japan)	2012	Retrospective	7	41.9	7/0	Arthroscopic CC stabilization with Endobutton and artificial ligament (+ washer in clavicle)	29
Motta et al^23^ (Italy)	2014	Retrospective	10	32	10/0	Arthroscopic CC stabilization with TightRope	24
Chen et al^6^ (Taiwan)	2014	Retrospective comparative	40	43.2	28/12	CC stabilization with Mersilene tape	38.2
Loriaut et al^20^ (France)	2015	Retrospective	21	33	14/7	Arthroscopic CC stabilization with TightRope	35
Kanchanatawan et al^15^ (Thailand)	2015	Retrospective	39	37.5	32/7	Modified CC stabilization with subcoracoid fiberwire sutures tight over 2 endobuttons	35.7
Choi et al^9^ (Korea)	2015	Retrospective	13	40.1	8/5	Open CC stabilization with double button (4) or suture anchor (9) and KW-tension band fragment fixation	14.1
Struhl & Wolfson^31^ (USA)	2016	Retrospective	6	43	4/2	Open CC stabilization with closed-looped double endobutton + suture fixation	40
Cano-Martínez et al^3^ (Spain)	2016	Retrospective	12	32.2	10/2	Open CC stabilization with Twin Tail TightRope	26
Cho et al^7^ (Korea)	2017	Retrospective	18	48.6	8/10	Open CC stabilization with TightRope	46
Cisneros et al^10^ (Spain)	2017	Retrospective	9	36	5/4	Arthroscopic CC stabilization with TightRope (+ fragment sutures)	49
Blake et al^2^ (USA)	2017	Retrospective	17	41	12/5	Arthroscopic CC stabilization with TightRope + fiberwire AC joint tension band	12
Sautet et al^28^ (France)	2018	Retrospective	14	34.6	10/4	Arthroscopic CC stabilization with subcoracoid suture and button (Dog Bone)	20
Xiong et al^33^ (China)	2018	Retrospective comparative	16	NR	NR	Arthroscopic double Endobutton fixation	57
Mochizuki et al^21^ (Japan)	2019	Retrospective	45	34.3	NR	Arthroscopic CC stabilization with Zip Tight (+ AC joint KW)	18.6
Yagnik et al^34^ (USA)	2019	Retrospective	18	55.5	14/4	Open CC stabilization with subcoracoid suture and button (Dog Bone) + allograft tendon augmentation (+ peek screw)	30.2
Sarda^27^ (UK)	2019	Retrospective	19	38	13/6	All suture modified under-coracoid-around-clavicle technique ± AC suturing	23
Levy et al^18^ (Australia)	2020	Retrospective comparative	30	NR	NR	CC stabilization with suture loop and transosseous sutures	12
		Total	421				

Shaded gray: Arthroscopic assisted coracoclavicular stabilization with buttons, with or without interfragmentary sutures or tension band; Nonshaded: Open coracoclavicular stabilization with buttons, subcoracoid sutures, mersilene tapes, cables or suture anchors.

### Characteristics of included studies

The total number of reported Neer IIb/Cho IIc distal clavicle fractures managed with a coracoclavicular stabilization technique in all studies was 421, ranging from 6 to 45 patients. In total, 139 (33%) patients received arthroscopic assisted treatment and 282 (67%) patients open techniques of coracoclavicular stabilization. There were 3 retrospective comparative studies comparing (a) arthroscopic coracoclavicular stabilization with buttons vs. hook plate vs. locking plate,^16^ (b) coracoclavicular stabilization with mersilene tapes vs. hook plate,^17^ and (c) coracoclavicular stabilization with suture loop and transosseous sutures vs. locking plate.^12^ In these three reports, only patients who underwent surgery with coracoclavicular stabilization techniques were included in our data synthesis. The mean age of patients was 39.1 years (32-55 years), the male-to-female ratio 3:1, and the mean follow-up period 30.2 months (4.6-57.3 months). Key characteristics of the 21 included studies are summarized in Table I.

We categorized the reported surgical techniques in 2 general types: arthroscopic-assisted coracoclavicular stabilization with buttons (Endobutton, TightRope, Zip Tight, Dog bone), with or without interfragmentary sutures/wires or with additional AC joint fixation with KW or tension band (8 studies), and open coracoclavicular stabilization with buttons, subcoracoid sutures, mersilene tapes, cables, or suture anchors (13 studies) (Table I). One arthroscopic study used an artificial ligament stabilized with a washer in the clavicle^18^ and one open technique study a tendon allograft stabilized with a peek screw in the clavicle.^19^ Supplementary internal fixation in addition to CC stabilization was used in 6 of 13 open technique studies in the form of interfragmentary sutures,^12^^,^^20^^,^^21^ interfragmentary wire,^22^ AC joint tension band with sutures,^23^ or KW^24^ and in 3 of 8 arthroscopic studies using either interfragmentary sutures,^25^ AC joint fixation with KW,^26^ or fiberwire.^27^

### Risk of bias assessment

All studies were retrospective with a high or moderate ROBINS-I overall risk of bias assessment. The heterogeneity of the operative techniques and the different evaluation methods prohibited us from performing a meta-analysis. Thus, our systematic review has a low level of evidence (IV).

### Effectiveness

Clinical results were reported with various clinical scores, including the Constant score (10 studies), ASES (5 studies), UCLA (6 studies), Oxford score (2 studies), DASH (4 studies), Karlsson’s criteria (1 study) and Modified Shoulder Rating Scale for Clavicle Fractures (1 study) (Table II). Nine studies used more than one score for clinical evaluation. The reported clinical results were very good to excellent in almost all studies; for example, in 10 of 11 studies using the Constant score, the mean values at the last follow-up evaluation were more than 90 points. In both arthroscopic and open techniques, the coracoclavicular stabilization with or without supplementary interfragmentary, or AC joint fixation, turned out to be an effective surgical technique.

**Table II:** Efficacy and safety of coracoclavicular stabilization surgical techniques in Neer IIB/Cho IIC fractures.

Author	N	Efficacy clinical score							Complications	
		ASES	CS	DASH	UCLA	Oxford score	Modified shoulder rating scale	Karlsson’s criteria	Major (n)	Minor (n)
Arthroscopic assisted coracoclavicular stabilization with buttons, with or without interfragmentary sutures or tension band										
Takase et al^32^	7	-	-	-	6 excellent, 1 good	-		-	None	None
Motta et al^23^	10	-	95 ± 0.73	-	-	-		-	None	Intraoperative fracture of coracoid lateral wall (revised immediately to a new Tightrope) (1)
Cisneros et al^10^	9	-	89.7 ± 8.5	11.9 ± 7	-	-		-	Nonunion (1)	Hardware prominence (1) Shoulder stiffness (1)
Blake et al^2^	17	90.1 ± 10.1	-	10.9 ± 11.1	-	-		-	Nonunion (3)	Early infection (irrigation) (1) Frozen shoulder (physiotherapy) (1) Prominent suture (1)
Sautet et al^28^	14	-	91% (85–95)∗	-	-	-		-	None	Delayed union (1) Hardware irritation (4) (2 were removed)
Xiong et al^33^	16	-	90.2 ± 12.2	-	-	-		-	Nonunion (1)	None
Mochizuki et al^21^	45	92.3 ± 3.2	94.1 ± 3.0	3.8 ± 2.8	-	-		-	None	Removal of KW at 8-12 weeks (1)
Loriaut et al^20^	21	-	94.8 ± 9.9	-	-	-		-	Nonunion (1)	ACJ arthritis (1) Heterotopic ossification (2) Adhesive capsulitis (1)
Total	139								6 (4.3%)	16 (11.5%)
Open coracoclavicular stabilization with buttons, subcoracoid sutures, mersilene tapes, cables, or suture anchors										
Chen et al^5^	11	-	-	-	-	-	16.3 (12–20)		None	Delayed union (1)
Shin et al^29^	19	-	94 (88–100)	-	-	-		-	Nonunion (1)	Delayed union (2) Clavicular erosions (2) Malunion (1) Slight loss of reduction (2)
Li et al^19^	29	-	-	-	-	-		A (72.4%) B (27.6%)	Breakage of wires (1)	None
Yang et al^35^	28	-	-	-	34 (29–35)	-		-	None	Frozen shoulder (1) Protrusion of suture node (removal under local) (1)
Chen et al^6^	40	-	-	-	46.9 (45–48)	47.2 (45–48)		-	None	Superficial infection (1) Frozen shoulder (1)
Kanchanatawan et al^15^	39	91.5 (75–100)	93.4 (72–100)	-	-	-		-	None	Superficial infection (1) Tunnel enlargements without buttons migration (9)
Choi et al^9^	13	-	94.7 (88–100)	-	31.3 (22–35)	-		-	Refracture at clavicle holes (1)	Clavicular erosion (1)
Struhl & Wolfson^31^	6	92.5 ± 15.4	93.2 ± 10.1	-	-	-		-	None	Wound break down (1)
Cano-Martínez et al^3^	12	-	95.5 ± 5.2	3.3 ± 4.4	-	-		-	None	CC calcifications (2) Hypertrophic scar (1) Patient with discomfort (1) Superficial infection (1)
Cho et al^7^	18	88.6 ± 19.3	-	-	31.3 (14–35)	-		-	Nonunion (1) Intraoperative coracoid fracture (converted to AC joint tension band) (1)	Delayed union (1) Shoulder stiffness (1) Subsidence of clavicular button (4)
Yagnik et al^34^	18	88.1 (82–94)	-	-	32.5 (31–34)	-		-	None	None
Sarda^27^	19	-	-	-	-	43		-	None	Mild osteolysis (1) Partial loss of reduction (1)
Levy et al^18^∗	30	-	-	1.8 (0–28)	-	-		-	None	None
Total	282								5 (1.77%)	38 (13.4%)

*ACJ*, acomioclavicular joint; *ASES*, American Shoulder and Elbow Surgeons Shoulder score; *CS*, Constant score; *UCLA*, UCLA shoulder rating scale.

∗ Weighted CS. All clinical efficacy scores are reported as mean (standard deviation) or mean (range).

### Safety

The overall major complication rate was 2.6% (8 nonunions, 1 coracoid fracture, 1 fracture between clavicular holes, and 1 cable breakage), whereas minor complications were present in 54 of 421 patients (12.8%) (Table II). More major complications were found in arthroscopic-assisted techniques (6 of 139 fractures; 4.3%) than the open ones (5 of 282 fractures; 1.8%). In contrast, fewer minor complications were found in arthroscopic techniques (16 of 139 fractures; 11.5%) compared with open techniques (54 of 282 patients; 13.4%). There were no cases of neurovascular compromise or deep infection. Thirteen studies (62%) reported no major complications at all.

## Discussion

The present systematic review of coracoclavicular stabilization techniques for Neer IIb and extralateral (IIc) fractures of the distal clavicle demonstrates acceptable effectiveness and safety. The reported clinical results were very good to excellent in almost all studies and the overall complication rate was 2.6%.

Treatment for unstable distal clavicle fractures is controversial and numerous techniques have been applied, which are generally divided into five main categories: hook plate, locking anatomic or T-plate, open or arthroscopic-assisted coracoclavicular fixation, interfragmentary/intramedullary fixation, and transacromial fixation with pins and/or tension band. The reported clinical outcomes and complication rates varied among different studies which have in general a retrospective design, a low level of evidence, and a small number of reported cases. Boonard et al^28^ performed a systematic review and network metanalysis in 2018, comparing postoperative shoulder function and complication rates between various fixation methods to identify the most effective and safe fixation technique for unstable distal clavicle fractures. Among ten comparative studies (505 fractures) and one randomized-controlled trial (42 fractures), the Constant-Murley scores for coracoclavicular fixation were significantly higher when compared with hook plate and tension band wiring. In contrast, a systematic review of eleven cohort studies (634 fractures), demonstrated no significant differences in the functional outcome and union rates between hook plate fixation, coracoclavicular stabilization, and locking plate fixation.^29^ However, hook plate fixation resulted in a higher Constant-Murley score compared with tension band wiring but was also associated with a higher complication rate compared with coracoclavicular stabilization and the locking plate. Finally, Oh et al^11^ in a systematic review on Neer type II fractures found that the complication rate was significantly higher with the use of the hook plate (40.7%) or tension band wiring (20.0%), compared with coracoclavicular (4.8%), intramedullary (2.4%), and interfragmentary fixation (6.3%). A limitation of this study was the uncertainty of whether type II fractures were constituted of mainly type IIa or IIb patterns, as most included studies did not specify the type II subclassification.

Special attention is required for some specific complications after coracoclavicular button fixation, including migration, slippage, coracoid or clavicle erosion, and button subsidence though the tunnels. This can lead to redislocation of the proximal clavicle and loss of fracture reduction. A similar problem has been encountered with the use of these techniques in acromioclavicular joint dislocations.^30^ The aforementioned complications may be attributed to the mispositioning of the tunnels and the excessive tension of the paired button bearings, which increase the force of slippage, especially when laid on the uneven face of the clavicle or the coracoid process. Furthermore, when the tension caused by the button on the clavicle or the coracoid process is too concentrated, this can lead to bone erosion. This phenomenon is usually encountered when a broad tunnel receives a button with a relatively small total area. Dog-ears, flat buttons, and two-tail systems (triple buttons) can solve this problem by distributing the tension forces evenly.^31^^,^^32^

We find that precise interpretation of distal clavicle fracture patterns can guide the surgeon in choosing the most appropriate surgical technique; however, interpretation of unstable types (IIa, IIb, IIc, and V) is inherently challenging.^3^, ^4^, ^5^^,^^10^ Neer’s classification has been widely used over the past decades to classify these fractures; however, as per Bishop et al,^33^ the interrater agreement is only fair for distal fragment size and type, moderate for stability and treatment approach, and slight for type IIb fractures. Similarly, Raurer et al found low interobserver and intraobserver agreement levels exhibited in all three classification systems that were studied (Neer, OTA, Jäger/Breitner), by two specialist groups (surgeons and radiologists), suggesting that all such classification systems are far from perfect and, arguably, of limited value.^34^ More recently though, Cho et al^5^ suggested a new classification system that considers both fracture displacement and stability, in addition to fracture location. This classification system showed moderate interobserver (ĸ = 0.434) and substantial intraobserver (ĸ = 0.644) reliability, while also it is designed to guide surgeons in choosing the most appropriate treatment option and implant type for each type of fracture; for example, coracoclavicular or transacromial fixation for extralateral IIc fractures.

We agree with Cho et al^5^ and Levy et al^12^ that the interpretation of the small distal fragment is difficult and a modification of the Neer classification paramount to include this rare type of fracture. Of even greater importance is the selection of an appropriate fixation technique for these unstable extralateral fractures, as the lateral fragment is particularly small and usually unable to hold traditional hardware. Cho et al^5^ suggested coracoclavicular stabilization techniques or transacromial intramedullary fixation for this type of fractures, whereas others have suggested traditional hardware (locking plates) but with additional coracoclavicular augmentation.^9^^,^^35^^,^^36^

The limitations of our systematic review lie with those of the included studies. All included studies were retrospective cohort, or case series, with a moderate to high risk of bias, deeming the level of evidence of our review as low. Furthermore, most studies only had a small sample size and a short follow-up period. Finally, although we aimed to synthesize our findings into two distinct fracture patterns (IIb and extralateral), this was not possible as most studies (20 of 21) combined the two in their reported findings.

## Conclusions

The present systematic review of coracoclavicular stabilization techniques for unstable Neer IIb and extralateral fractures of the distal clavicle demonstrates promising clinical outcomes, including effectiveness and safety. Treatment options for this type of fracture such as locking plate fixation are limited owing to their small size that cannot support firm internal fixation. Open or arthroscopic coracoclavicular loop techniques, augmented in some cases with interfragmentary sutures or tension band techniques, seem to provide adequate fixation of the fracture with excellent clinical outcomes and a low complication rate. We support the previously proposed modification of the Neer classification to include this unique type of unstable extralateral fracture (type IIc) to allow for targeted surgical management.

## Disclaimers

Funding: No funding was disclosed by the authors.

Conflicts of interest: The authors, their immediate families, and any research foundations with which they are affiliated have not received any financial payments or other benefits from any commercial entity related to the subject of this article.
